# Strain Dependence of Hysteretic Giant Magnetoimpedance Effect in Co-Based Amorphous Ribbon

**DOI:** 10.3390/ma12132110

**Published:** 2019-06-30

**Authors:** Michał Nowicki, Piotr Gazda, Roman Szewczyk, Andriy Marusenkov, Anton Nosenko, Vasyl Kyrylchuk

**Affiliations:** 1 Warsaw University of Technology, Institute of Metrology and Biomedical Engineering, 02-495 Warsaw, Poland; 2 Lviv Center of the Institute of Space Research, 79060 Lviv, Ukraine; 3 V. Kurdyumov Institute for Metal Physics of NAS of Ukraine, 03142 Kyiv, Ukraine

**Keywords:** GMI, soft magnetic materials, stress-impedance, magnetomechanical sensor

## Abstract

The significant strain dependence of the hysteretic Giant Magnetoimpedance (GMI) effect in a Co_67_Fe_3_Cr_3_B_12_Si_15_ amorphous alloy in a low magnetizing field is presented. A simplistic test stand capable of continuous measurements of GMI characteristics under the influence of strain is detailed. Based on the results, a stress-impedance (SI) sensor is proposed, with a gauge factor similar to semiconductor strain gauges but more robust. An effective method of minimizing external magnetic field influence on the SI effect is given.

## 1. Introduction

The Giant Magnetoimpedance effect (GMI) [1] has been heavily researched [2,3,4,5,6], mainly as a basis for novel magnetic field sensors [7], which were actually developed even before the discovery of the ‘giant’ version of the magnetoimpedance (MI) effect [8,9]. Many of the works are oriented towards maximization of the GMI coefficient [10], that is, the relative impedance change of the investigated sample under the influence of an external magnetizing field. It currently reaches 600% and more for cobalt-based amorphous wires and amorphous ribbons but is still far from the theoretical limit [11]. There are many factors influencing the resulting characteristics, with these being mainly the sample chemical composition, crystal/amorphous structure, magnetic domain structure, geometrical parameters, driving current amplitude and frequency, and even non-magnetic coatings [12]. There is also the magnetomechanical effect of impedance change due to mechanical stress in the sample, known as the stress-impedance (SI) [13,14,15,16]. This is caused by the Villari effect, which is the change of magnetic permeability of ferromagnetic material due to internal stresses [17]. Thus, the GMI and SI effects are closely connected, and influence each other.

One of the key factors connected to a high GMI coefficient is the high relative magnetic permeability of the sample, as it directly corresponds to skin depth for high frequency (HF) currents, which are the main physical mechanism behind the GMI effect. High-permeability cobalt-based amorphous ribbons are typical candidates for GMI applications. Thus, a Co_67_Fe_3_Cr_3_B_12_Si_15_ amorphous alloy developed previously for magnetic fluxgates [18] was tested. It was chosen due to its material high permeability and low noise exhibited in fluxgates.

The investigated material exhibited a very high GMI coefficient when measured with an HF impedance bridge in a high-field GMI test stand described elsewhere [19]. There was significant asymmetry between the peak values of its double-peak characteristic. This is a known behavior which can be due to a number of factors, such as DC biasing current, AC current amplitude and frequency, and external field inhomogeneity, etc. [20,21,22]. The characteristics however are highly unstable when measured point-by-point with an occasional ‘check’ for saturation GMI value due to apparent magnetic hysteresis, and due to mechanical interference. This has called for the development of a low-field GMI test stand capable of continuous measurements of GMI characteristics, with a symmetrical magnetizing field waveform and sample strain control during the tests. The results have shown significant GMI hysteresis but also significant strain dependence for the investigated alloy. This is an undesired effect for GMI magnetic field sensors; however, it can be utilized in SI strain gauges. 

Thus, a strain sensing method is suggested which takes advantage of the strain-dependent part of the GMI characteristic. The gauge factor of the proposed sensor falls between 270 in the high sensitivity region and 120 near saturation, which is a similar value to semiconductor strain gauges.

Stress-impedance strain gauges have been reported previously as having gauge factors as high as 1300 but in these the magnetic field influence was often not controlled or set to a fixed value, and the SI ratio/strain characteristics were highly nonlinear or even non-monotonous [23,24,25]. 

The proposed solution is virtually immune to weak (~200 μT) stray magnetic fields due to the continuous ‘sweeping’ of the sample with the *H* field from the magnetizing coil and measurement of the strain-dependent part of the GMI curve only. 

## 2. Materials and Methods

### 2.1. Sample Preparation

The ribbon samples used in this investigation were made of a Co_67_Fe_3_Cr_3_B_12_Si_15_ amorphous alloy. Utilized samples were developed as the modified version of the Co-based amorphous alloy MELTA^®^ MM-5Co with nearly zero magnetostriction, a Curie temperature of *T_C_* = 460 K, very high relative permeability (*µ_max_ ~* 250,000), and saturation induction *B* = 0.42 T [18]. The material’s electrical resistivity was measured as 1.86 × 10^−6^ Ω·m. It was produced using a rapid-quenching technique. The material was originally developed for magnetic fluxgate cores and similar devices [26]. The results of low magnetizing field hysteresis measurements are presented in [27].

In order to induce magnetic anisotropy and raise relative permeability, the material for the samples was annealed for 1 h at 440 °C in an oxidation inhibiting CO_2_ atmosphere. For this annealing temperature the ribbon was mainly amorphous, as the differential scanning calorimetry (DSC) measured temperature of crystallization onset is 535°C [27]. At the cooling stage (approximately at 250 °C), a transverse DC field of 40 kA/m was applied until the core reached 100 °C, after which it was removed from the oven for cooling. It induced a slight transverse magnetic anisotropy in the ribbons. The cooling rate was in the range of 0.4–1.3 °C/min. The samples prepared at the different cooling rates in the mentioned range had similar values of the initial permeability.

The samples used were in ribbon form and were 1.5mm wide, 50 mm long, and 20 μm thick. In order to achieve a good electrical connection and secure the mechanical mounting during straining, samples were soldered to the sample holder electrical contacts with a standard low melting point solder. Because the amorphous alloy used was not wetted by the solder, copper was electrodeposited on the sample ends with help of a solution of blue vitriol (copper(II) sulfate). The DC resistance of the sample was 3.1 Ω.

### 2.2. Measurement Method

**Figure 1:**
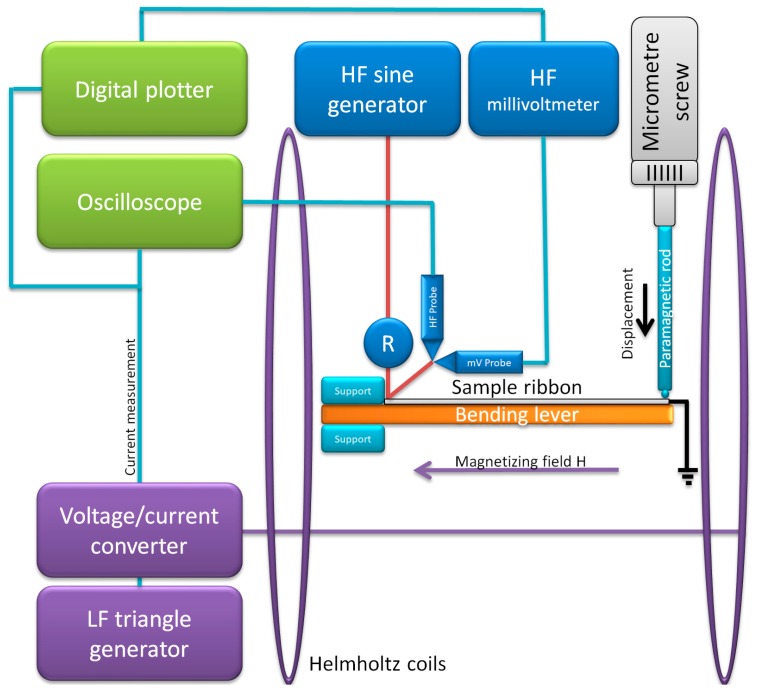
Schematic diagram of the developed Giant Magnetoimpedance (GMI) measurement system. Legend: HF, high frequency; LF, low frequency.

The measurements were carried out in a custom-designed low-field GMI measurement system (Figure 1). This system was based on the voltage divider principle, utilizing measurement of the HF voltage drop *U* on the ribbon sample, which was connected in series with a non-inductive standard resistor [28]. 

The HF sample driving signal was applied using an HF sinewave generator (Channel 1 of SDG 2082X, Siglent, Helmond, The Netherlands), and measured either by a 1 GHz digital millivoltmeter (В3-52/1, Машприборинторг, Moscow, Russia), or a 1 GHz HF demodulator probe (V40.25, Meratronik, Warsaw, Poland) connected to an oscilloscope (SDS1074CFL, Siglent, Helmond, The Netherlands). The voltage applied was set to 400 mV RMS and the frequencies were chosen as 1, 5, 10, and 20 MHz.

A magnetizing *H* field was generated by a pair of Helmholtz coils powered by a voltage-current converter (RMD-2b, WUT, Warsaw, Poland), which was controlled by a low frequency (LF) triangle wave generator (Channel 2 of SDG 2082X, Siglent, Helmond, The Netherlands). The magnetizing waveform during tests was set to 20 mHz and a ±420 A/m amplitude. This prevented the system (mostly millivoltmeter) from having an additional ‘time lag’ hysteresis and still allowed for measurement of full characteristics in 50 s. 

The rectified HF voltage drop *U* on the sample and driving current signal were fed to the oscilloscope and digital plotter system (HP7090A Measurement Plotting System, Hewlett-Packard, Palo Alto, CA, USA), which served as the data acquisition and visualization system. The impedance of the sample was calculated using Ohm’s law with the measured voltage drop *U*, the HF resistance value of the resistor, and the applied voltage amplitude. The magnetizing field *H* was calculated from the magnetizing current signal and Helmholtz coil dimensions.

The system was further equipped with a bending lever sample holder, which, together with a micrometer screw, allowed for controlled straining of the ribbon being tested. The strain was calculated from the displacement of the free end of the lever based on a test stand presented in [23,29].

(1) $$\epsilon =\frac{3dh\left(L-x/2\right)}{2L^{3}}$$

In the above equation ϵ is strain; *d* is the deflection of the free end of the lever; *h* is the thickness of the lever; *L* is the length of the lever; and *x* is the length of the ribbon sample.

In the presented research the parameters were *h* = 1 mm and *L = x* = 55 mm.

For full deflection of *d* = 2 mm the strain was ϵ = 495 × 10^−6^.

## 3. Results and Discussion

**Figure 2:**
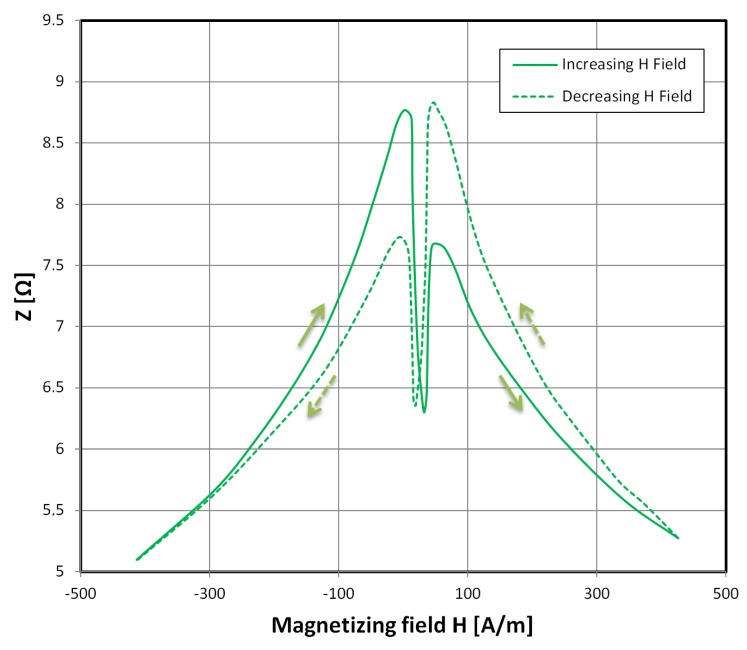
Hysteretic dependence of sample impedance (for a 1 MHz driving signal) on a changing magnetizing field *H* applied longitudinally. The green line indicates the characteristics for an increasing *H* field and the green dashed line indicates the characteristics for a decreasing *H* field.

Exemplary results of impedance *Z* versus magnetizing field *H* characteristics for the MELTA^®^ MM-5Co alloy are presented in Figure 2. As can be seen, the characteristics measured one way (with increasing magnetizing field values) had significant asymmetry, and this is mirrored for the decreasing field values (a switch in the asymmetry direction is visible), forming the distinct GMI hysteretic response. The right-side shift of the peaks is due to the uncompensated Earth’s magnetic field component.

GMI hysteresis in low magnetizing fields has been reported in previous works [30,31,32,33,34] but it is most often neglected and not reported at all. It has even been assumed that GMI curves are anhysteretic, and magnetic field sensors are made under this assumption, which seems justified for most utilized materials. 

**Figure 3:**
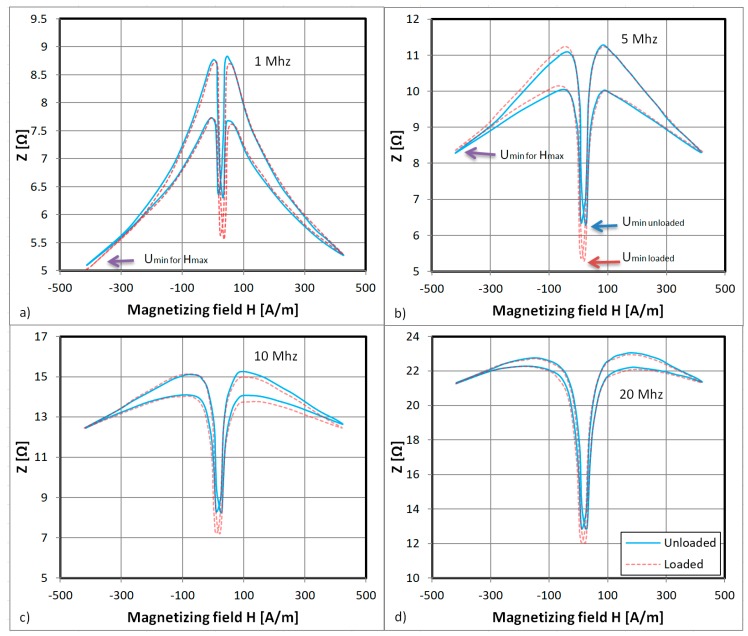
Dependence of sample impedance on the magnetizing field *H* applied longitudinally for (**a**) a 1 MHz driving signal; (**b**) a 5 MHz driving signal; (**c**) a 10 MHz driving signal; and (**d**) a 20 MHz driving signal. The blue line indicates the characteristic for zero strain and the red dashed line indicates the characteristic for fully applied strain. The blue and red arrows indicate the *U_min_* voltage drop minima for the strained and unstrained samples.

In Figure 3 the strain dependence of the GMI curve of the investigated sample is given. It is an important factor influencing GMI sensor response [35]. As can be seen, for a 1 MHz driving signal (Figure 3a), the negative peaks near the zero field are significantly lower for the strained sample (2 mm deflection of the bending lever and ϵ = 495 × 10^−6^). The strain–dependent *U* signal minima, however, have higher values than the minima for the maximum magnetizing field, which inhibits simple utilization of this magnetomechanical effect.

**Figure 4:**
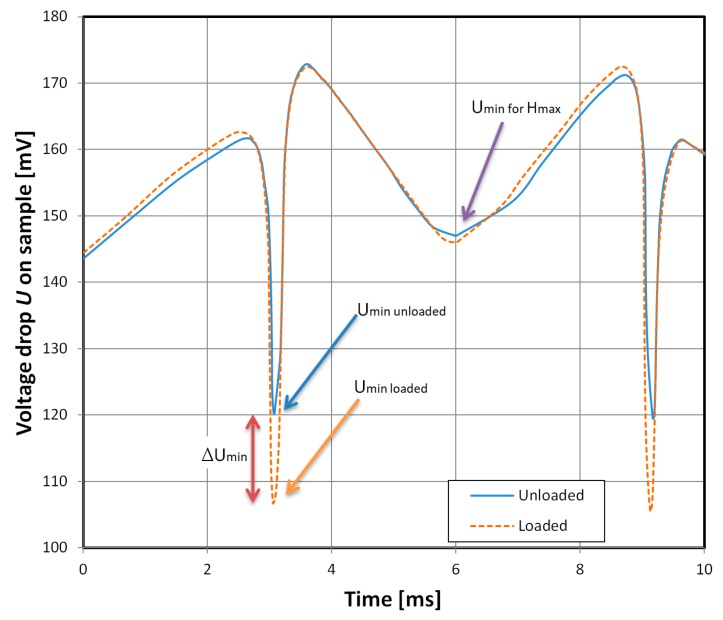
Measured HF voltage drop *U* signal for a 5 MHz, 400 mV RMS driving signal. The minimum of the voltage drop signal *U_min_* (corresponding to arrows in Figure 3b) is the strain-dependent part of the measured waveform.

In Figure 3b the driving signal frequency is raised to 5 MHz and the negative peaks of the characteristics are lower than its sides. This means that the minima of the voltage drop *U_min_* for the sample (corresponding to the arrow positions) are unambiguously related to the strain-dependent negative peaks of the GMI characteristic (the measured voltage drop U is shown in Figure 4).

For the 10 and 20 MHz driving signals (Figure 3c,d), the hysteresis is slowly vanishing and the peaks of this double-peak characteristic are leaving the investigated low-field region. This is caused by the known-frequency-dependence of the GMI characteristics, where for a higher exciting frequency the peaks manifest at higher magnetizing field values [36]. 

The “double-peak” GMI characteristics consist of the superposition of three effects [36]. The transverse susceptibility of the sample changes with an external DC magnetizing field perpendicular to the easy axis of magnetization due to magnetic domain movement (a single-peak, parabolic characteristic) and due to magnetization rotation (a double peak characteristic with maxima for *H* field values corresponding to the anisotropy field *H_k_*) [36]. There is also smaller hysteretic influence of the DC magnetizing field *H* parallel to the easy magnetization axis. The local minimum near the zero field is due to the magnetization rotation in the sample and there is significant strain influence on this effect observed in the presented GMI characteristics. 

The strain dependence of the samples was further investigated with the help of a bending lever sample holder and micrometer screw setting the deflection of its free end. For these measurements, the driving signal was set to 5 MHz and 400 mV RMS, as this frequency allowed for unambiguous measurement of the strain-sensitive part of the characteristic. It corresponded to *U_min_*, the minimum of the voltage drop signal (Figure 4). The magnetizing field *H* was set to 43 Hz and ±420 A/m amplitude. The measurements of *U_min_* changes were taken with the oscilloscope and HF demodulator probe, which allowed for fast response of the measurement system to the strain change.

**Figure 5:**
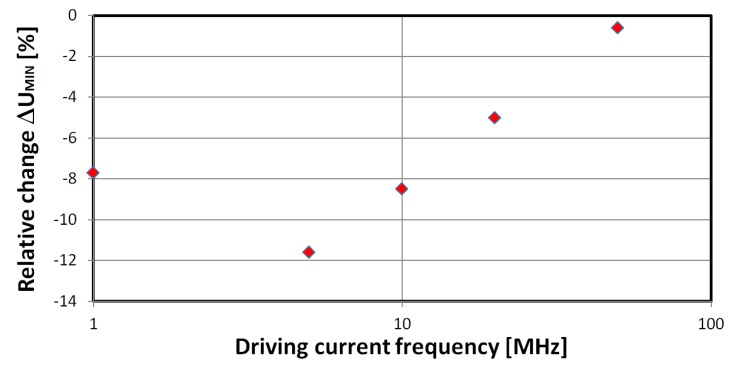
Dependence of measured ∆*U_min_*, the minimum of the voltage drop signal, under full strain, on the driving current frequency.

The 5 MHz driving signal was chosen due to the higher relative change ∆*U_min_* of the *U_min_* signal. A graph of relative change of ∆*U_min_* signal dependence on driving current frequency is presented in Figure 5. Lower frequencies were omitted due to the voltage drop *U* minima for maxima of the applied *H* field. For the investigated material, only the near-zero *H* field part of the GMI characteristic is interesting from a strain-sensing point of view. Furthermore, the *U_SI_* signal of the proposed sensor, which is directly proportional to ∆*U_min_*, was measured with help of a demodulator probe, negative peak detector circuit, and voltmeter. The strain was increased to the critical value (ϵ = 1240 × 10^−6^), above which the sample was prone to breaking.

**Figure 6:**
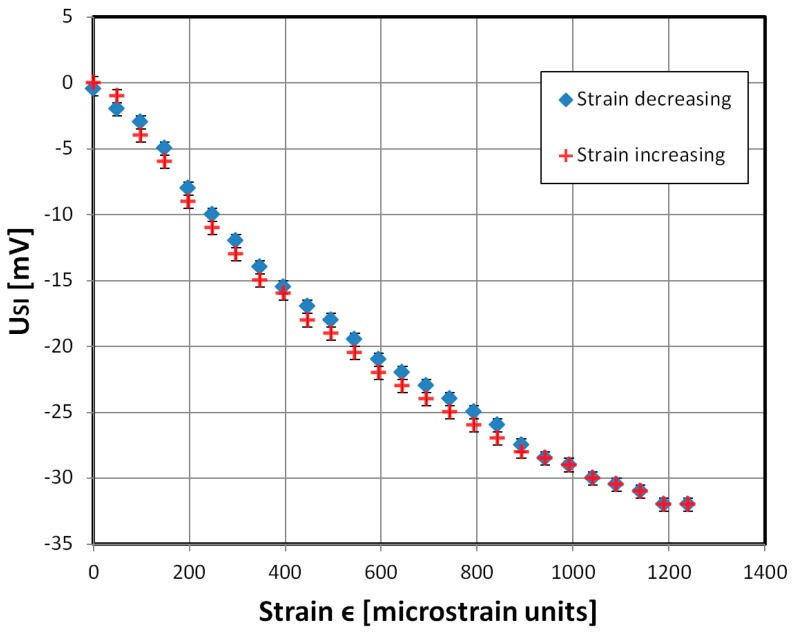
Dependence of measured *U_SI_* signal, the relative change of the minimum of the voltage drop signal (corresponding to the amber arrow in Figure 4), on the sample strain, for increasing (red cross) and decreasing (blue rhomb) strain values. A 5 MHz, 400 mV RMS driving signal was used. The highest SI ratio ΔZ/Z = −15%.

The characteristic of the *U_SI_* signal for the sample on strain is presented in Figure 6 and shows some hysteresis for the increasing-decreasing strain measurement series, which is typical of magnetoelastic systems [37]. This is mainly due to the magnetoelastic hysteresis of the material. There are proposed solutions for compensating for similar problems [38,39]. The gauge factor however equals 120 for critical strain (ϵ = 1240 × 10^−6^) and 270 in the high sensitivity region for ϵ = 248 × 10^−6^. This was calculated with the following formula:

(2) F= (ΔZ/Z)ϵ

In this formula, *F* is the gauge factor and ΔZ/Z is the SI ratio for the strain used. It is 60–135 times higher than for standard metallic strain gauges and similar to the semiconductor strain gauges (which have F = 130–200).

Stress-impedance strain gauges have had reported gauge factors as high as 1300, but the magnetic field influence has often not been controlled, and the SI ratio/strain characteristics have been highly nonlinear or even non-monotonous [26,38,39,40].

The main advantage of the presented material working as an SI sensor is omitting the need for external magnetic field compensation with DC magnetizing field *H* biasing. The right solution is AC *H* field sweeping in the low-field range with the help of an additional coil, and measurement of *U_min_*, the minimum of the voltage drop signal. This would allow for SI measurements unaffected by relatively weak external magnetic fields, such as the Earth’s geomagnetic field, or stray fields induced by ferromagnetic objects.

## 4. Conclusions

The hysteretic GMI effect on a Co_67_Fe_3_Cr_3_B_12_Si_15_ amorphous alloy was presented in this work, with this effect explaining repeatability errors and asymmetry in GMI characteristics obtained by point-by point methods. One of the most interesting features of this particular composition, which is responsible for magnetic impedance hysteresis, has been described previously as high order magnetic anisotropy and it has been analyzed using magnetic domains and theoretical model calculations [41]. This hints at the need for high resolution symmetrical (field increasing-field decreasing) measurements of GMI characteristics in novel magnetic materials and systems. This is especially important for samples exhibiting asymmetry in their characteristics, such as uneven peaks for “double-peak” materials. 

The strain dependence of the GMI minimum near zero *H* field (the minimum of the double-peak characteristic) is due to strain influence on magnetization rotation near the zero-field region. This presents the possibility of high gauge factor stress-impedance force sensors development. 

The proposed strain sensing technique can be implemented with simple measurement of the negative peak of the demodulated voltage drop for the presented amorphous ribbon. Due to *H* field sweeping it is robust to relatively weak external magnetic fields (~200 μT) which would otherwise shift the working point of the sensor. It is especially important for stress-impedance sensors which are typically highly sensitive to external DC magnetic fields.

## References

[1] Panina L., Mohri K., Bushida K., Noda M. (1994). Giant magneto-impedance and magneto-inductive effects in amorphous alloys. J. Appl. Phys..

[2] Yu J., Zhou Y., Cai B., Xu D. (2000). Giant magneto-impedance effect in amorphous magnetostrictive FeSiB thin films. J. Magn. Magn. Mater..

[3] Morikawa T., Nishibe Y., Yamadera H., Nonomura Y., Takeuchi M., Taga Y. (1997). Giant magneto-impedance effect in layered thin films. IEEE Trans. Magn..

[4] Panina L.V., Mohri K. (2000). Magneto-impedance in multilayer films. Sens. Actuators A Phys..

[5] Zhukova V., Blanco J.M., Ipatov M., Gonzalez J., Churyukanova M., Zhukov A. (2018). Engineering of magnetic softness and giant magnetoimpedance effect in Fe-rich microwires by stress-annealing. Scr. Mater..

[6] Chobaomsup V., Jantaratana P., Boonyongmaneerat Y. (2018). Effects of Current Pulsation on Magnetic Properties and Giant Magnetoimpedance of Electrodeposited NiFe Coatings on Cu Wires. Mater. Sci. Forum.

[7] Hauser M., Kraus L., Ripka P. (2001). Giant magnetoimpedance sensors. IEEE Instrum. Meas. Mag..

[8] Harrison E.P., Rowe H. (1938). An impedance magnetometer. Proceed. Phys. Soc..

[9] Turney G.L., Cousins G.E. (1938). A portable direct-reading magnetometer. J. Sci. Instrum..

[10] Knobel M., Pirota K.R. (2002). Giant magnetoimpedance: Concepts and recent progress. J. Magn. Magn. Mater..

[11] Corte-León P., Zhukova V., Ipatov M., Blanco J.M., Gonzalez J., Zhukov A. (2019). Engineering of magnetic properties of Co-rich microwires by joule heating. Intermetallics.

[12] Kraus L. (2003). GMI modeling and material optimization. Sens. Actuators A Phys..

[13] Shen L.P., Uchiyama T., Mohri K., Kita E., Bushida K. (1997). Sensitive stress-impedance micro sensor using amorphous magnetostrictive wire. IEEE Trans. Magn..

[14] Tejedor M., Hernando B., Sánchez M.L., Prida V.M., Vázquez M. (1998). The torsional dependence of the magneto-impedance effect in current-annealed Co-rich amorphous wires. J. Phys. D Appl. Phys..

[15] Beato-López J.J., Vargas-Silva G., Pérez-Landazábal J.I., Gómez-Polo C. (2018). Giant stress-impedance (GSI) sensor for diameter evaluation in cylindrical elements. Sens. Actuators A Phys..

[16] Nematov M.G., Salem M.M., Adam A., Ahmed M., Panina L.V., Morchenko A.T. (2017). Effect of stress on magnetic properties of annealed glass-coated Co_71_Fe_5_B_11_Si_10_Cr_3_ amorphous microwires. IEEE Trans. Magn..

[17] Mihalca I., Ercuta A., Ionascu C. (2003). The Villari effect in Fe–Cr–B amorphous ribbons. Sens. Actuators A Phys..

[18] Nosenko V.K., Maslov V.V., Kirilchuk V.V., Kochkubey A.P. (2008). Some industrial applications of amorphous and nanocrystalline alloys. J. Phys. Conf. Ser..

[19] Gazda P., Nowicki M., Szewczyk R. (2018). Influence of alloy composition on GMI effect in amorphous ribbons. AIP Conf. Proc..

[20] Makhnovskiy D.P., Panina L.V., Mapps D.J. (2000). Asymmetrical magnetoimpedance in as-cast CoFeSiB amorphous wires due to ac bias. Appl. Phys. Lett..

[21] Phan M.H., Yu S.C., Kim C.G., Vázquez M. (2003). Origin of asymmetrical magnetoimpedance in a Co-based amorphous microwire due to dc bias current. Appl. Phys. Let..

[22] Ciureanu P., Khalil I., Melo L.G.C., Rudkowski P., Yelon A. (2002). Stress-induced asymmetric magneto-impedance in melt-extracted Co-rich amorphous wires. J. Magn. Magn. Mater..

[23] Mao X.H., Zhou Y., Chen J.A., Yu J.Q., Cai B.C. (2003). Giant magnetoimpedance and stress-impedance effects in multilayered FeSiB/Cu/FeSiB films with a meander structure. J. Mater. Res..

[24] Mohri K., Uchiyama T., Shen L.P., Cai C.M., Panina L.V. (2001). Sensitive micro magnetic sensor family utilizing magneto-impedance (MI) and stress-impedance (SI) effects for intelligent measurements and controls. Sens. Actuators A Phys..

[25] Cobeno A.F., Zhukov A., Blanco J.M., Larin V., Gonzalez J. (2001). Magnetoelastic sensor based on GMI of amorphous microwire. Sens. Actuators A Phys..

[26] Marusenkov A. (2017). Possibilities of further improvement of 1s fluxgate variometers. Geosci. Instrum. Methods Data Syst..

[27] Nowicki M., Szewczyk R., Charubin T., Marusenkov A., Nosenko A., Kyrylchuk V. (2018). Modeling the Hysteresis Loop of Ultra-High Permeability Amorphous Alloy for Space Applications. Materials.

[28] Gazda P., Kachniarz M., Szudarek M. (2017). Test stand for investigating of giant magneto-impedance. International Conference Automation.

[29] Zhou Y., Mao X.H., Chen J.A., Ding W., Gao X.Y., Zhou Z.M. (2005). Stress-impedance effects in layered FeSiB/Cu/FeSiB films with a meander line structure. J. Magn. Magn. Mater..

[30] Sinnecker J.P., Tiberto P.A.O.L.A., Kurlyandskaia G.V., Sinnecker E.H.C.P., Vázquez M., Hernando A. (1998). Hysteretic giant magneto impedance. J. Appl. Phys..

[31] Ipatov M., Zhukova V., Zhukov A., Gonzalez J., Zvezdin A. (2010). Low-field hysteresis in the magnetoimpedance of amorphous microwires. Phys. Rev. B.

[32] Ipatov M., Zhukova V., Gonzalez J., Zhukov A. (2014). Magnetoimpedance hysteresis in amorphous microwires induced by core–shell interaction. Appl. Phys. Lett..

[33] Béron F., Valenzuela L.A., Knobel M., Melo L.G., Pirota K.R. (2012). Hysteretic giant magnetoimpedance effect analyzed by first-order reversal curves. J. Magn. Magn. Mater..

[34] Zhukova V., Blanco J.M., Ipatov M., Churyukanova M., Taskaev S., Zhukov A. (2018). Tailoring of magnetoimpedance effect and magnetic softness of Fe-rich glass-coated microwires by stress-annealing. Sci. Rep..

[35] Nabias J., Asfour A., Yonnet J.-P. (2017). The Impact of Bending Stress on the Performance of Giant Magneto-Impedance (GMI) Magnetic Sensors. Sensors.

[36] Knobel M., Vázquez M., Kraus L. (2003). Giant magnetoimpedance. Handb. Magn. Mater..

[37] Kraus L., Švec P. (2003). Magnetoelastic hysteresis of amorphous ribbons. J. Appl. Phys..

[38] Smith R.C. (2001). Inverse compensation for hysteresis in magnetostrictive transducers. Math. Comput. Model..

[39] Oppermann K., Arminger B.R., Zagar B.G. Smart hysteresis compensation of a magneto-elastic force sensor based on Terfenol-D. In Proceeding of 2010 IEEE Instrumentation and Measurement Technology Conference (I2MTC).

[40] Arai K.I., Muranaka C.S., Yamaguchi M. (1994). A new hybrid device using magnetostrictive amorphous films and piezoelectric substrates. IEEE Trans. Magn..

[41] Kurlyandskaya G.V., Vazquez M., Munoz J.L., Garcia D., McCord J. (1999). Effect of induced magnetic anisotropy and domain structure features on magnetoimpedance in stress annealed Co-rich amorphous ribbons. J. Magn. Magn. Mater..

